# Effects of empagliflozin in different phases of diabetes mellitus-related cardiomyopathy: a prospective observational study

**DOI:** 10.1186/s12872-021-02024-3

**Published:** 2021-04-29

**Authors:** Satoshi Oka, Takahiko Kai, Katsuomi Hoshino, Kazunori Watanabe, Jun Nakamura, Makoto Abe, Akinori Watanabe

**Affiliations:** Department of Cardiology, Fujieda Municipal General Hospital, Surugadai 4-1-11, Fujieda, Shizuoka 426-8677 Japan

**Keywords:** Diabetes mellitus-related cardiomyopathy, Heart failure, Sodium–glucose co-transporter 2 inhibitor, Left ventricular dysfunction, Left ventricular global longitudinal strain

## Abstract

**Background:**

Diabetes mellitus-related cardiomyopathy (DMCMP), defined as left ventricular (LV) dysfunction caused by hyperglycemia in the absence of coronary artery disease, leads to heart failure (HF). Previous studies have shown that treatment with sodium-glucose co-transporter 2 inhibitor (SGLT2i) reduces the risk of exacerbation of HF. The beneficial effects of SGLT2i on HF depend not only on indirect actions such as osmotic diuresis but also on direct actions on the myocardium, leading to improvements in LV function. However, it remains unclear whether SGLT2i treatment is equally effective in any phase of DMCMP. The aim of this observational study was to compare the efficacy of SGLT2i treatment on LV dysfunction between early and advanced DMCMP.

**Methods:**

Thirty-five symptomatic non-ischemic HF patients with LV ejection fraction > 40% and type 2 diabetes mellitus (T2DM) treated with empagliflozin (EMPA group) and 20 controls treated without SGLT2i were enrolled. According to the myocardial extracellular volume fraction (ECV), a reliable marker of cardiac fibrosis quantified by cardiac magnetic resonance, the EMPA group was further divided into early DMCMP (n = 16, ECV ≤ 30%) and advanced DMCMP (n = 19, ECV > 30%) groups and followed up prospectively. Echocardiography was performed at baseline and after 12 months. LV function assessed as LV global longitudinal strain (LVGLS) and the ratio of early diastolic mitral inflow velocity to early diastolic mitral annular velocity (E/e′) were compared.

**Results:**

ECV was strongly correlated with T2DM duration (r^2^ = 0.65, *p* < 0.001). At baseline, each group had a similar background. After 12 months, the EMPA group, especially the early DMCMP group, showed remarkable improvements in LVGLS (ΔLVGLS: 2.9 ± 3.0% (EMPA) vs. 0.6 ± 2.2% (controls), *p* = 0.005, and 4.6 ± 1.5% (early DMCMP) vs. 1.6 ± 3.3% (advanced DMCMP), *p* = 0.003) and E/e′ (ΔE/e′: − 1.5 ± 4.7 vs. − 0.3 ± 3.0, *p* = 0.253, and − 3.4 ± 5.5 vs. − 0.1 ± 3.5, *p* = 0.043).

**Conclusions:**

The positive effects of empagliflozin on LV dysfunction were more remarkable in early than in advanced DMCMP. Early intervention of SGLT2i for DMCMP may be preferable.

## Background

Type 2 diabetes mellitus (T2DM) is an important risk factor for the development of cardiovascular disease and heart failure (HF) [1]. Diabetes mellitus-related cardiomyopathy (DMCMP), which manifests as left ventricular (LV) dysfunction that occurs independently of coronary artery disease and hypertension [2], has been attracting attention as a cause of HF. Hyperglycemia drives LV dysfunction and remodeling through the progression of microvascular endothelial dysfunction, myocardial injury, and interstitial fibrosis, which lead to DMCMP [3]. Decreased LV global longitudinal strain (LVGLS) and the increased ratio of early diastolic mitral inflow velocity to early diastolic mitral annular velocity (E/e′) are observed as signs of LV dysfunction from the early phase of DMCMP [4, 5]. If LV remodeling progresses, DMCMP develops to symptomatic HF with preserved ejection fraction (HFpEF) or HF with reduced ejection fraction (HFrEF) [3].

Several mega-trials have shown that treatment with sodium-glucose co-transporter 2 inhibitor (SGLT2i) reduces the risk of major adverse cardiovascular events, including exacerbation of HF [6–8]. Furthermore, it was recently shown that SGLT2i treatment was associated with lowering the risk of cardiovascular death and hospitalization for HFrEF consistently from the early to late periods after the start of administration, regardless of the presence or absence of T2DM [9, 10]. Thus, the beneficial effects of SGLT2i treatment on HF are explained not only by their indirect actions, such as glycemic control or osmotic diuresis, but also by their direct actions on the myocardium, which lead to improvements in LV function [11, 12]. One example of direct action is inhibition of the sodium-hydrogen exchanger (NHE), which may in turn lead to a reduction in myocardial injury, fibrosis, and LV dysfunction [13].

However, it remains unclear whether SGLT2i treatment is equally effective in any phase of DMCMP. The aim of this study was to compare the efficacy of SGLT2i treatment on LV dysfunction between the early and advanced phases of DMCMP.

## Methods

### Patients

This was a prospective observational study conducted at a single center. Consecutive symptomatic HF patients with T2DM who were hospitalized in Fujieda Municipal General Hospital (Japan) were screened for eligibility. The diagnosis of T2DM was based on the World Health Organization criteria [14]. After a cardiac assessment, patients diagnosed with DMCMP with LV ejection fraction (EF) > 40%, and treated with starting the administration of empagliflozin (at a dose of 10 mg daily) were enrolled as the EMPA group. Contrast-enhanced cardiac magnetic resonance (CMR) was performed in all patients in the EMPA group, and their myocardial extracellular volume fraction (ECV), a reliable marker of cardiac fibrosis, was evaluated. According to previous reports [15, 16], global ECV > 30% was considered elevated with advanced replacement myocardial fibrosis. Therefore, the EMPA group was further divided into the early DMCMP group (global ECV ≤ 30%) and advanced DMCMP group (global ECV > 30%). Meanwhile, to confirm the efficacy of empagliflozin on LV dysfunction, DMCMP patients with LVEF > 40% who had never used SGLT2i were also included as a control group after a cardiac assessment with plain CMR. All participants were prospectively followed up.

Exclusion criteria were as follows: (1) age < 20 or > 80 years; (2) in-hospital death; (3) New York Heart Association (NYHA) class I or brain natriuretic peptide (BNP) < 100 pg/mL; (4) LVEF ≤ 40%; (5) coronary artery disease, hypertensive heart disease, or other cardiomyopathy; (6) valvular or congenital heart disease; (7) LVGLS within normal range (absolute value ≥ 18%) [4]; (8) type 1 diabetes mellitus or insulin-dependent T2DM with a C-peptide immunoreactivity index < 0.8 [17] or use of insulin; (9) newly diagnosed T2DM (< 1 year) or no antidiabetic medications before the start of administration of empagliflozin; (10) previous use of SGLT2i; (11) persistent arrhythmia; (12) pacemaker implantation; (13) contraindication for CMR (implanted metallic objects, allergy to contrast media, or bronchial asthma); (14) estimated glomerular filtration rate (eGFR) ≤ 30 mL/min/1.73 m^2^; (15) malignant tumor or inflammatory disease; (16) pregnancy; (17) refusal to provide informed consent, and (18) history of myocardial infarction, cerebral infarction, pancreatitis, or hospitalization for HF. To exclude patients with coronary artery disease, coronary angiography was performed in all participants. Patients with ≥ 90% coronary artery stenosis were excluded. Patients with 75% stenosis were also excluded if a > 10% ischemic area matched with angiography results was proven by myocardial perfusion scintigraphy. Regarding hypertensive heart disease, patients with diastolic blood pressure ≥ 90 mmHg were excluded [3]. Regarding other cardiomyopathies, patients with regional LV wall motion abnormalities, late gadolinium enhancement (LGE), excessive LV dilatation (LV end-diastolic volume index > 97 mL/m^2^ [3]), and excessive LV hypertrophy (LV myocardial mass index > 69 g/m^2^ for women or > 91 g/m^2^ for men [18]) as evaluated by CMR were not included. Because contrast-enhanced CMR was not performed in the control group patients, they were exempted from LGE assessment.

### Outcomes

The primary outcome was improvement in LV function, defined as changes in LV systolic function assessed as LVGLS and diastolic function assessed as E/eʹ between baseline and 12 months after starting the administration of empagliflozin. The secondary outcomes were the NYHA class after 12 months and the changes in glycated hemoglobin (HbA1c) and BNP levels between baseline and after 12 months.

### Anthropometrics and blood tests

At the time of enrollment, age, sex, height, body weight, blood pressure, and heart rate of all participants were recorded. NYHA class and serum hemoglobin, HbA1c, sodium, eGFR, and BNP levels at admission were used as baseline data. Fasting C-peptide and plasma glucose levels were measured along with a hematocrit measurement at the time of CMR, and the C-peptide immunoreactivity index was calculated using the following formula: fasting C-peptide/fasting plasma glucose × 100. Serum HbA1c and BNP levels were also measured routinely at 12 months.

### Ultrasonic echocardiography

**Fig. 1:**
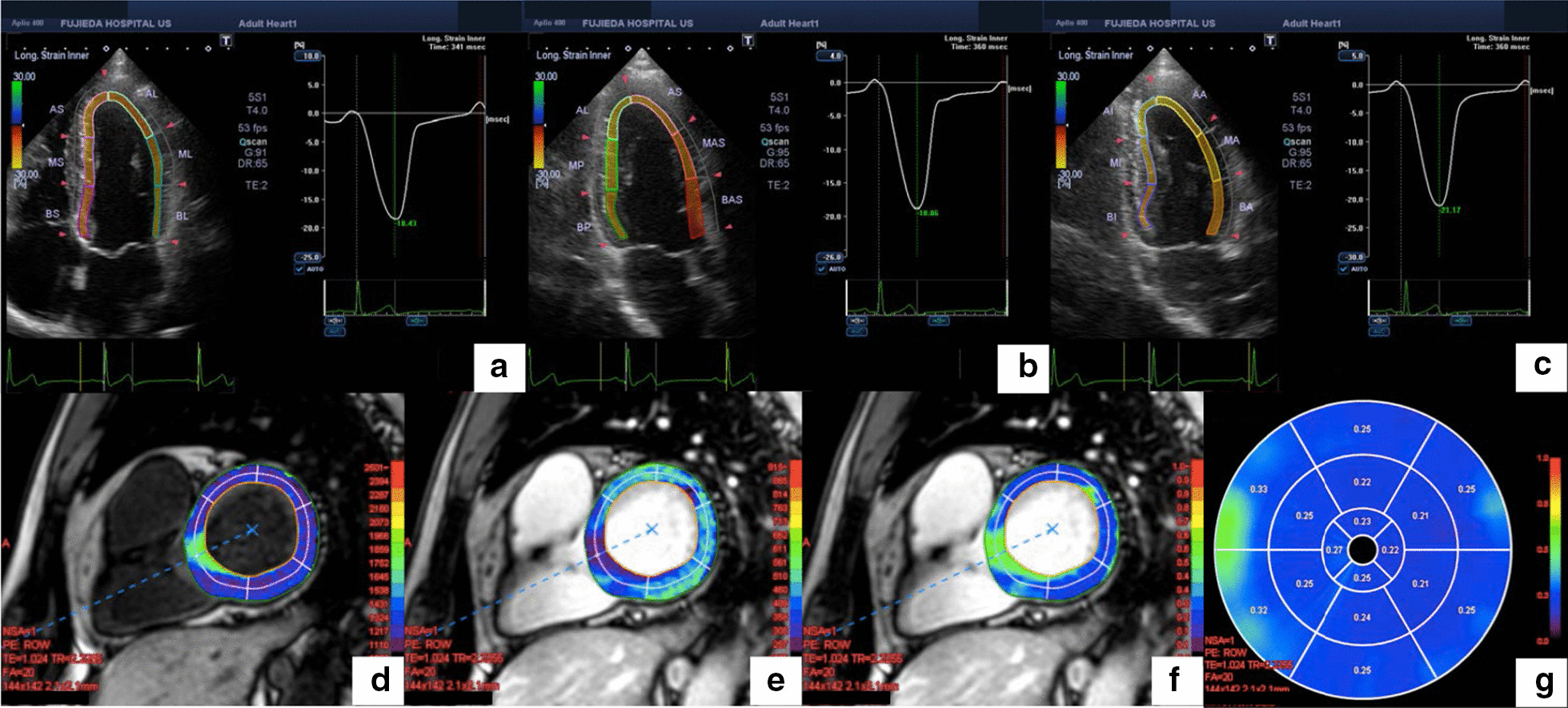
Example of assessment of left ventricular global longitudinal strain (LVGLS) and extracellular volume fraction (ECV). Apical four- (**a**), three- (**b**), and two-chamber (**c**) views of two-dimensional speckle-tracking echocardiography imaging are shown. LVGLS was calculated by averaging the values from these results. Maps of the LV basal short-axis segment with a modified Look-Locker inversion recovery sequence, native T1 mapping (**d**), post-contrast T1 mapping (**e**), and calculated ECV mapping (**f**) are shown. Global ECV value was calculated by averaging the values of the American Heart Association 16-segment model (**g**)

Ultrasonic echocardiography was performed at baseline and after 12 months using an Aplio 400^®^ (Canon Medical Systems Corporation, Tochigi, Japan) by two cardiac ultrasonographers who were blinded to the patients’ backgrounds. Two-dimensional gray-scale cine loops from three consecutive heartbeats were obtained at end-expiratory apnea from standard parasternal and apical views. According to the guidelines of the American Society of Echocardiography/European Association of Cardiovascular Imaging [19], standard echocardiographic measurements were performed. LVEF was measured using the modified Simpson method. The E-wave velocity was measured using pulsed-wave Doppler recording from the apical four-chamber view. Spectral pulsed-wave Doppler-derived e′ was obtained by averaging the septal and lateral mitral annulus, and the E/e′ was calculated to obtain an estimate of LV filling pressure. LVGLS was measured using two-dimensional speckle-tracking echocardiography. Speckle-tracking strain was analyzed using the 2D Wall Motion Tracking Application^®^ software (Canon Medical Systems Corporation, Tochigi, Japan). While maximizing the frame rate, the LV endocardial border was traced manually at the end-diastolic frame. The software automatically tracked the myocardium throughout the cardiac cycle. The peak values of six segmental longitudinal strains were obtained from the apical four-, three-, and two-chamber views, and the LVGLS was calculated by averaging the values (Fig. 1).

### CMR scanning protocol

All CMR exams were performed using a 3.0-T scanner (Ingenia^®^; Philips, Eindhoven, Netherlands) with a 32-element cardiac receiver coil. Vector-electrocardiogram-gated standard steady-state free precession cine sequences were acquired in short axes covering the whole LV and long-axis (four-, three-, and two-chamber) views. LGE images were acquired 10 min post-contrast (Gadovist^®^ 0.1 mmol/kg) injection. T1 maps were generated before and 15 min after gadolinium contrast injection using a modified Look-Locker inversion recovery sequence [20] during breath-holding in end-expiration to produce 11 raw images with increasing inversion times (TI, 100–5000 ms) in an LV short-axis view (TR/TE, 2.20/1.02 ms; flip angle, 20°). Blood samples were taken for hematocrit determination within 24 h before the scan. All maps were analyzed using Ziostation2^®^ version 2.9 2–2 (Ziosoft, Tokyo, Japan). Myocardial T1 values and ECV were determined by drawing regions of interest in each segment of the LV slice according to the American Heart Association 16-segment model (Fig. 1). ECV values were calculated according to the following formula: ECV = (1 − HCT) × (1/T1 value_myocardium post_ − 1/T1 value_myocardium pre_)/(1/T1 value_blood post_ − 1/T1 value_blood pre_). The global ECV was calculated by averaging the values of the 16 segments.

### Statistical analysis

We included data from all patients in the analysis of baseline characteristics and outcomes according to the intention-to-treat principle. Normally distributed continuous variables are expressed as mean and standard deviation. Levene’s test showed that T2DM duration, eGFR, BNP, left atrial dimension, and LV end-diastolic dimension were not distributed normally. These variables are expressed as median and interquartile range. Student’s *t*-test or Mann–Whitney U test was used to compare differences between the two groups, where appropriate. The differences between two matched samples were compared using a paired *t*-test. A simple linear regression analysis was performed to evaluate the correlations. All statistical tests were two-tailed, and values of *p* < 0.05 were considered to indicate statistical significance. IBM SPSS Statistics^®^ version 19.0 (SPSS, Chicago, IL, USA) was used for statistical analyses.

## Results

### Baseline characteristics

**Fig. 2:**
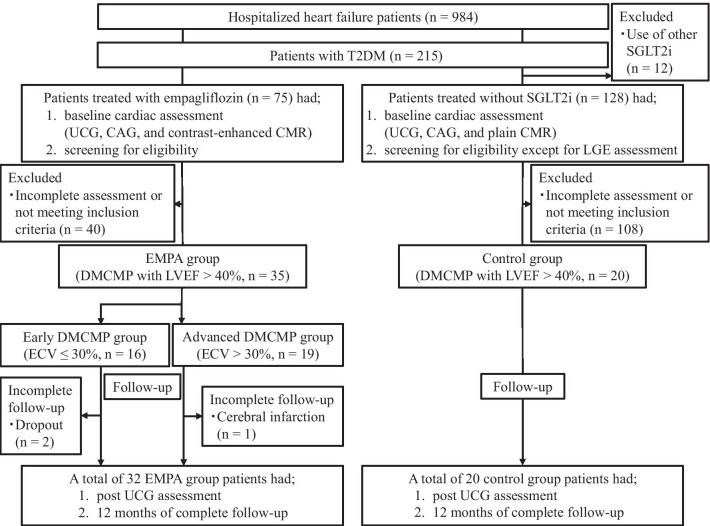
Enrollment and follow-up. Flow diagram shows the recruitment and follow-up process of this study. Abbreviations: CAG, coronary angiography; CMR, cardiac magnetic resonance; DMCMP, diabetes mellitus-related cardiomyopathy; ECV, extracellular volume fraction; LGE, late gadolinium enhancement; LVEF, left ventricular ejection fraction; SGLT2i, sodium-glucose co-transporter 2 inhibitor; T2DM, type 2 diabetes mellitus; UCG, ultrasonic echocardiography

A total of 984 HF patients were hospitalized between April 1, 2017, and June 1, 2019, and 215 of these patients with T2DM were screened for eligibility. A flow diagram of patient recruitment for this study is illustrated in Fig. 2. A total of 35 DMCMP patients treated with empagliflozin were enrolled as the EMPA group. Meanwhile, 20 control patients treated without SGLT2i also participated. At baseline, both groups had similar backgrounds (Table 1).

**Table 1:** Baseline characteristics of all participants

	EMPA group (n = 35)	Control group (n = 20)	*p* value
*Backgrounds*			
Age (years old)	66.4 ± 10.0	65.7 ± 10.5	0.794
Male gender (%)	26 (74.3)	15 (75.0)	0.954
Body mass index (kg/m^2^)	24.2 ± 4.4	24.5 ± 4.5	0.755
NYHA class	3.1 ± 0.8	2.8 ± 0.8	0.208
Systolic blood pressure (mmHg)	122.8 ± 16.1	121.8 ± 19.0	0.828
Diastolic blood pressure (mmHg)	77.6 ± 8.6	74.7 ± 6.4	0.195
Heart rate (bpm)	79.2 ± 10.4	80.9 ± 11.1	0.583
Hypertension (%)	29 (82.9)	16 (80.0)	0.796
Dyslipidemia (%)	29 (82.9)	15 (75.0)	0.493
T2DM duration (months)	48 (24–103)	44 (31–47)	0.895
Hemoglobin (g/dl)	14.1 ± 2.4	13.4 ± 3.2	0.381
HbA1c (%)	8.2 ± 1.5	7.9 ± 0.7	0.339
Sodium (mEq/l)	139.1 ± 3.2	139.8 ± 2.0	0.375
eGFR (ml/min/1.73m^2^)	73.7 (64.7–77.8)	70.2 (57.9–77.9)	0.489
BNP (pg/ml)	498 (304–709)	525 (314–811)	0.766
*UCG characteristics*			
LAD (mm)	44 (39–46)	44 (40–45)	0.916
LVDd (mm)	51 (48–54)	50 (48–55)	0.435
LVEF (%)	51.8 ± 10.5	52.5 ± 10.7	0.827
LVGLS (absolute value) (%)	7.2 ± 2.8	7.6 ± 3.0	0.642
E/e′	12.9 ± 5.0	12.9 ± 5.1	0.962
* CMR characteristics*			
LVEDV index (ml/m^2^)	61.5 ± 17.1	60.5 ± 19.7	0.836
LVM index (g/m^2^)	49.6 ± 11.7	51.6 ± 14.3	0.570
*Medications*			
β blocker (%)	18 (51.4)	11 (55.0)	0.803
ACEi (%)	4 (11.4)	4 (20.0)	0.395
ARB (%)	26 (74.3)	14 (70.0)	0.737
ARNI (%)	0 (0.0)	0 (0.0)	–
Loop diuretics (%)	28 (80.0)	18 (90.0)	0.479
MRA (%)	5 (14.3)	4 (20.0)	0.590
Tolvaptan (%)	0 (0.0)	2 (10.0)	0.163
SGLT2i (%)	35 (100)	0 (0.0)	–
Metformin (%)	12 (34.3)	4 (20.0)	0.250
DPP4i (%)	28 (80.0)	17 (85.0)	0.493
GLP-1 agonist (%)	2 (5.7)	0 (0.0)	0.160
Thiazolidine (%)	2 (5.7)	0 (0.0)	0.160
Sulfonylurea (%)	2 (5.7)	1 (5.0)	0.913
Grinide (%)	0 (0.0)	0 (0.0)	–
α-GI (%)	6 (17.1)	4 (20.0)	0.796

Data are means ± SD for normally distributed data and medians and interquartile ranges for non-normally distributed data, or n (%). All statistical tests were 2-tailed, and *p* < 0.05 was considered significant (*)

NYHA, New York Heart Association; T2DM, type 2 diabetes mellitus; HbA1c, glycated hemoglobin; eGFR, estimated glomerular filtration rate; BNP, brain natriuretic peptide; UCG, ultrasonic echocardiography; LAD, left atrial dimension; LVDd, left ventricular end-diastolic dimension; LVEF, left ventricular ejection fraction; LVGLS, left ventricular global longitudinal strain; E/e', ratio of early diastolic mitral inflow velocity to early diastolic mitral annular velocity; CMR, cardiac magnetic resonance; LVEDV, left ventricular end-diastolic volume; LVM, left ventricular mass; ACEi, angiotensin-converting enzyme inhibitor; ARB, angiotensin II receptor blocker; ARNI, angiotensin receptor neprilysin inhibitor; MRA, mineralocorticoid receptorantagonist; SGLT2i, sodium-glucose co-transporter 2 inhibitor; DPP4i, dipeptidyl peptidase-4 inhibitor; GLP-1, glucagon like peptide-1; α-GI, alpha-glucosidase inhibitor

**Fig. 3:**
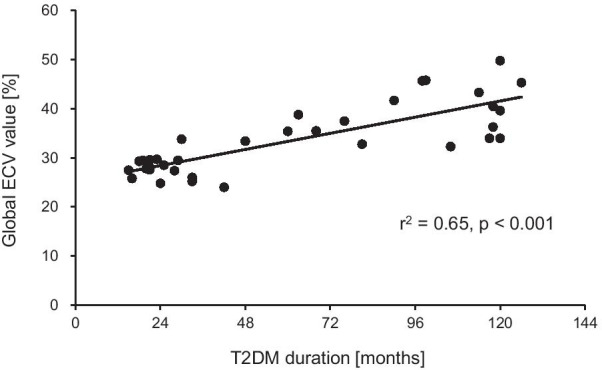
Scatter plot of extracellular volume fraction (ECV) value and type 2 diabetes mellitus (T2DM) duration. The basic linear regression line shows a strong correlation between the global ECV value (y-axis) and T2DM duration (x-axis)

The EMPA group patients were further divided into early DMCMP (n = 16, global ECV: 27.5 ± 1.9%) and advanced DMCMP (n = 19, global ECV: 38.7 ± 5.3%) groups. The baseline characteristics of the two groups are summarized in Table 2. There were no significant differences in age, sex, NYHA class, HbA1c, BNP, LVGLS, and E/e′. However, LVEF was significantly lower in the advanced DMCMP group than in the early DMCMP group (55.9 ± 10.0% vs. 48.4 ± 9.8%, *p* = 0.032). The T2DM duration of the advanced DMCMP group was significantly longer than that of the early DMCMP group (22 [19–28] vs. 99 [72–118] months, *p* < 0.001). Interestingly, the global ECV value was strongly correlated with T2DM duration (r^2^ = 0.65, *p* < 0.001, Fig. 3).

**Table 2:** Baseline characteristics of EMPA group

	Early DMCMP group (n = 16)	Advanced DMCMP group (n = 19)	*p* value
*Backgrounds*			
Age (years old)	63.1 ± 10.4	69.2 ± 9.0	0.070
Male gender (%)	14 (87.5)	12 (63.2)	0.097
Body mass index (kg/m^2^)	25.3 ± 5.0	23.1 ± 3.8	0.147
NYHA class	2.9 ± 0.7	3.3 ± 0.8	0.146
Systolic blood pressure (mmHg)	127.0 ± 18.6	119.3 ± 13.0	0.159
Diastolic blood pressure (mmHg)	76.9 ± 7.8	78.1 ± 9.1	0.698
Heart rate (bpm)	80.4 ± 9.1	78.2 ± 11.6	0.519
Hypertension (%)	13 (81.3)	16 (84.2)	0.823
Dyslipidemia (%)	13 (81.3)	16 (84.2)	0.823
T2DM duration (months)*	22 (19–28)	99 (72–118)	< 0.001
Hemoglobin (g/dl)	14.7 ± 1.8	13.7 ± 2.7	0.241
HbA1c (%)	8.4 ± 1.7	8.1 ± 1.3	0.586
Sodium (mEq/l)	139.4 ± 2.3	138.8 ± 3.9	0.560
eGFR (ml/min/1.73m^2^)	73.7 (73.0–77.8)	64.7 (58.4–77.2)	0.185
BNP (pg/ml)	459 (383–709)	498 (285–807)	0.894
C-peptide immunoreactivity index	1.3 ± 0.2	1.1 ± 0.3	0.062
Hematocrit (%)	42.1 ± 3.8	40.8 ± 5.7	0.432
*UCG characteristics*			
LAD (mm)	43 (41–46)	44 (39–46)	0.987
LVDd (mm)	50 (48–53)	52 (49–58)	0.280
LVEF (%) *	55.9 ± 10.0	48.4 ± 9.8	0.032
LVGLS (absolute value) (%)	7.9 ± 2.4	6.7 ± 3.0	0.207
E/e′	13.2 ± 6.1	12.6 ± 3.8	0.694
*CMR characteristics*			
LVEDV index (ml/m^2^)	56.7 ± 14.4	65.6 ± 18.4	0.125
LVM index (g/m^2^)	55.9 ± 10.0	48.4 ± 9.8	0.698
Native T1 value (ms)*	1224.6 ± 47.4	1365.8 ± 52.7	< 0.001
ECV (%)	27.5 ± 1.9	38.7 ± 5.3	–
*Medications*			
β blocker (%)	7 (43.8)	11 (57.9)	0.419
ACEi (%)	2 (12.5)	2 (10.5)	0.860
ARB (%)	12 (75.0)	14 (73.7)	0.932
Loop diuretics (%)	13 (81.3)	15 (78.9)	0.823
MRA (%)	3 (18.8)	2 (10.5)	0.503
Empagliflozin (%)	16 (100)	19 (100)	–
Metformin (%)	4 (25.0)	8 (42.1)	0.297
DPP4i (%)	13 (81.3)	15 (78.9)	0.870
GLP-1 agonist (%)	0 (0.0)	2 (10.5)	0.163
Thiazolidine (%)	0 (0.0)	2 (10.5)	0.163
Sulfonylurea (%)	1 (6.3)	1 (5.3)	0.904
α-GI (%)	1 (6.3)	5 (26.3)	0.109

Data are means ± SD for normally distributed data and medians and interquartile ranges for non-normally distributed data, or n (%). All statistical tests were 2-tailed, and *p* < 0.05 was considered significant (*). ECV denotes extra-cellular volume fraction. Other abbreviations as in Table 1

Finally, A total of 52 patients had 12 months of complete follow-up (Fig. 2).

### Primary outcomes

**Fig. 4:**
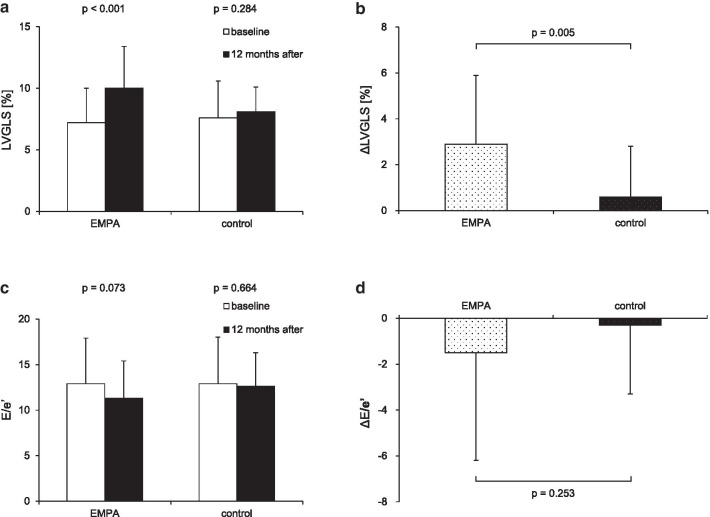
Results of primary outcomes: a comparison between EMPA and control groups. Left ventricular global longitudinal strain (LVGLS; absolute value) and the ratio of early diastolic mitral inflow velocity to early diastolic mitral annular velocity (E/e′) at baseline and 12 months after enrollment are shown in (**a**) and (**c**). The primary outcomes, changes in LVGLS and E/e′ between 12 months (ΔLVGLS and ΔE/e′) are shown in (**b**) and (**d**)

After 12 months, the EMPA group showed greater improvements in LVGLS than the control group (ΔLVGLS: 2.9 ± 3.0% vs. 0.6 ± 2.2%, *p* = 0.005, Fig. 4). Although not significant, a positive effect of empagliflozin on E/e′ was also observed (ΔE/e′: − 1.5 ± 4.7 vs. − 0.3 ± 3.0, *p* = 0.253, Fig. 4).

**Fig. 5:**
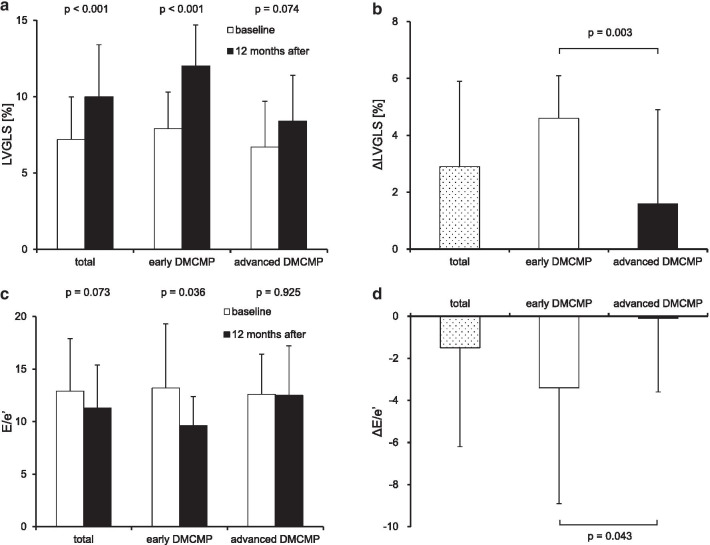
Results of primary outcomes of EMPA group: a comparison between early and advanced DMCMP groups. Left ventricular global longitudinal strain (LVGLS; absolute value) and the ratio of early diastolic mitral inflow velocity to early diastolic mitral annular velocity (E/e′) at baseline and 12 months after administration of empagliflozin are shown in (**a**) and (**c**). The primary outcomes, changes in LVGLS and E/e′ between 12 months (ΔLVGLS and ΔE/e′) are shown in (**b**) and (**d**). Improvements in LVGLS and E/eʹ are more remarkable in the early DMCMP group than in the advanced DMCMP group

Furthermore, the early DMCMP group showed more remarkable improvements in both LVGLS (ΔLVGLS: 4.6 ± 1.5% vs. 1.6 ± 3.3%, *p* = 0.003) and E/e′ (ΔE/e′: − 3.4 ± 5.5 vs. − 0.1 ± 3.5, *p* = 0.043) than the advanced DMCMP group (Fig. 5).

### Secondary outcomes

There were no significant differences between the EMPA and control groups, and between the early and advanced DMCMP groups in NYHA class after 12 months (1.3 ± 0.4 vs. 1.3 ± 0.5, *p* = 0.699, and 1.2 ± 0.4 vs. 1.3 ± 0.5, *p* = 0.755, Table 3) and the changes in HbA1c and BNP between baseline and after 12 months (ΔHbA1c: − 1.3 ± 1.5% vs. − 0.9 ± 0.5%, *p* = 0.214, and − 1.6 ± 1.5% vs. − 1.0 ± 1.4%, *p* = 0.249, ΔBNP: − 305 [197–504] pg/mL vs. − 302 [188–574] pg/mL, *p* = 0.910, and − 305 [201–400] pg/mL vs. − 398 [143–537] pg/mL, *p* = 0.594, Table 3).

**Table 3:** Secondary outcomes

	EMPA group	Control group	*p* value	Early DMCMP group	Advanced DMCMP group	*p* value
*NYHA class*						
12 months	1.3 ± 0.4	1.3 ± 0.5	0.699	1.2 ± 0.4	1.3 ± 0.5	0.755
*HbA1c* (*%*)						
12 months	7.1 ± 0.7	7.0 ± 0.6	0.473	7.0 ± 0.6	7.2 ± 0.7	0.527
ΔHbA1c	− 1.3 ± 1.5	− 0.9 ± 0.5	0.214	− 1.6 ± 1.5	− 1.0 ± 1.4	0.249
*BNP* (*pg/ml*)						
12 months	122 (106–165)	168 (127–240)	0.087	113 (106–160)	143 (106–163)	0.605
ΔBNP	− 305 (197–504)	− 302 (188–574)	0.910	− 305 (201–400)	− 398 (143–537)	0.594

Data are means ± SD for normally distributed data and medians and interquartile ranges for non-normally distributed data, or n (%)

All statistical tests were 2-tailed, and *p* < 0.05 was considered significant (*). Abbreviations as in Table 1

## Discussion

The present study showed positive effects of empagliflozin on LV functional parameters. Furthermore, the improvements were more remarkable in the early DMCMP group than in the advanced DMCMP group. HF parameters, such as NYHA class and BNP, were equally improved in both the early and advanced DMCMP groups.

### Characteristics of DMCMP

Hyperglycemia causes LV dysfunction, which leads to the development of DMCMP [3]. LV diastolic dysfunction is a classical LV functional abnormality observed in the preclinical phase of DMCMP [5]. LV longitudinal myocardial dysfunction has also been reported as one of the earliest markers of LV dysfunction in DMCMP [4]. If LV remodeling progresses, DMCMP develops into symptomatic HF showing restrictive HFpEF or dilated HFrEF phenotypes. Phenotype-specific pathophysiological mechanisms have recently been proposed for LV dysfunction and remodeling consisting of coronary microvascular endothelial dysfunction, interstitial fibrosis, and myocardial hypertrophy in HFpEF and cardiomyocyte cell death and extensive replacement fibrosis in HFrEF [3].

In this study, symptomatic HF patients with T2DM were enrolled. Thus, although we named the group of patients with ECV ≤ 30% as the early DMCMP group, they were not strictly in the early phase. Their ECV values were as high as those reported in a previous report of patients with T2DM and normal LV function [21], but their LVGLS and E/eʹ were relatively worse than those reported in other studies targeting patients with T2DM and stable HF [11, 12]. The baseline LVEF was lower in the advanced DMCMP group than in the early DMCMP group; thus, the advanced DMCMP group patients might have a nearly dilated HFrEF phenotype. Their ECV values were very high and suggested extensive replacement fibrosis.

### Impact of SGLT2i on LV functional parameters

In line with previous reports using dapagliflozin [11, 12], the administration of empagliflozin also improved LV functional parameters such as LVGLS and E/e′. A previous study showed that dapagliflozin was more effective in improving LVGLS in T2DM patients with HFpEF than HFrEF [12]. Furthermore, our research targeting DMCMP with the non-HFrEF phenotype revealed that empagliflozin treatment showed greater improvements in LVGLS and E/e′ in the early phase than in the advanced phase. The potential advantage of starting the administration of SGLT2i before the progression of LV remodeling among T2DM patients is suggested.

### Mechanisms of direct cardiac effects

It is hypothesized that the direct cardiac effects of SGLT2i depend on a reduction in intracellular sodium by inhibiting NHE-1, which is expressed in the heart and vasculature [13]. In patients with T2DM and HF, the activity of NHE-1 is markedly enhanced. This increase facilitates the accumulation of intracellular sodium, which stimulates the reverse activity of the sodium-calcium exchanger, leading to an increase in intracellular calcium and myocardial injury [22]. The inhibition of NHE-1 reduces intracellular sodium and calcium concentrations, increases mitochondrial calcium, which restores mitochondrial function and a redox state, activates ATP production in the failing heart, and thus improves LV function [13]. In animal models, SGLT2i treatment, via the inhibition of NHE-1, reduces myocardial injury and fibrosis, slows the progression of DMCMP, and improves systolic and diastolic function [23–25]. These findings suggest that empagliflozin promotes reverse LV remodeling; thus, the lesser the degree of myocardial injury and fibrosis, the greater is the extent to which LV function could be restored by SGLT2i treatment.

Considering that there were no significant intergroup differences in ΔHbA1c, the reverse LV remodeling through the inhibition of NHE-1 was independent of the main effect of SGLT2i: glycemic control by blocking glucose reabsorption through SGLT2. Side effects, such as osmotic diuresis and inhibition of NHE, may be the reason why SGLT2i treatment is associated with lowering the risk of HF exacerbation regardless of the presence or absence of T2DM. The composite of direct and indirect cardiac actions of SGLT2i could improve HF parameters even in patients with advanced DMCMP.

### Clinical implications

LV longitudinal myocardial dysfunction and diastolic dysfunction are clinically important markers observed from the preclinical phase of DMCMP [4, 5], leading to HF. In the present study, symptomatic HF patients with LVEF > 40% and T2DM were enrolled and evaluated. As a result, treatment with empagliflozin improved LV functional parameters assessed as LVGLS and E/e′, which were more remarkable in the earlier phase of DMCMP. The phase progression was correlated with T2DM duration. These are clinically important findings indicating a potential benefit of early intervention with SGLT2i in HF patients with T2DM.

### Study limitations

This study has some limitations. First, this was a small observational study conducted at a single center. Therefore, several biases were possible. For instance, the advanced DMCMP group characteristics, including lower baseline LVEF, could have affected various outcomes. Although the EMPA group tended to have more remarkable improvements in E/eʹ than the control group, the difference was not significant, in contrast to a previous report [11]. We consider that the small study number and population differences, having more advanced DMCMP patients with higher baseline E/eʹ and treatment-resistance, might have affected the result. Given the study limitations, empagliflozin may have a strong positive effect on LVGLS because it was shown even in the population. In addition, there were no significant intergroup differences in the use of other medications, but the medication such as *β*-blocker could also have affected the result. Although a multivariate analysis adjusted for baseline characteristics should be performed to evaluate the effective factors for improving LV function, it was not possible because of the limited number of cases. Second, contrast-enhanced CMR was not carried out in the control group patients. Due to a lack of ECV evaluation in the control group, assessments with an interaction test were not possible. Furthermore, a myocardial biopsy was not performed in this study. Although we performed coronary angiography and CMR to increase the diagnostic accuracy of DMCMP, the possibility that patients with other cardiomyopathies were still included cannot be denied, especially in the control group assessed without LGE. Third, we performed only a short-term assessment of LV function. If the follow-up period was longer, LV functional parameters might have improved further in the advanced DMCMP group.

## Conclusions

Empagliflozin had positive effects on LV function that were more remarkable in the early DMCMP group with normal ECV values than in the advanced DMCMP group with elevated ECV values. The ECV increase was strongly correlated with T2DM duration. Thus, early SGLT2i administration for patients with HF and T2DM may be preferable.

## Data Availability

Not applicable.

## Abbreviations

BNP: Brain natriuretic peptide
CMR: Cardiac magnetic resonance
DMCMP: Diabetes mellitus-related cardiomyopathy
ECV: Extracellular volume fraction
EF: Ejection fraction
eGFR: Estimated glomerular filtration rate
E/e′: Ratio of early diastolic mitral inflow velocity to early diastolic mitral annular velocity
GLS: Global longitudinal strain
HbA1c: Glycated hemoglobin
HF: Heart failure
HFpEF: Heart failure with preserved ejection fraction
HFrEF: Heart failure with reduced ejection fraction
LGE: Late gadolinium enhancement
LV: Left ventricular
NHE: Sodium-hydrogen exchanger
NYHA: New York Heart Association
SGLT2i: Sodium-glucose co-transporter 2 inhibitor
T2DM: Type 2 diabetes mellitus
